# Health Technology Assessment: Global Advocacy and Local Realities: Comment on "Priority Setting for Universal Health Coverage: We Need Evidence-Informed Deliberative Processes, Not Just More Evidence on Cost-Effectiveness"

**DOI:** 10.15171/ijhpm.2016.118

**Published:** 2016-08-29

**Authors:** Kalipso Chalkidou, Ryan Li, Anthony J. Culyer, Amanda Glassman, Karen J. Hofman, Yot Teerawattananon

**Affiliations:** ^1^Institute of Global Health Innovation, Imperial College London, London, UK.; ^2^Department of Economics & Related Studies and Centre for Health Economics, University of York, York, UK.; ^3^Center for Global Development, Washington, DC, USA.; ^4^School of Public Health, Faculty of Health Sciences, University of the Witwatersrand, Johannesburg, South Africa.; ^5^Health Intervention and Technology Assessment Program (HITAP), Nonthaburi, Thailand.

**Keywords:** Deliberation, Cost-Effectiveness Analysis (CEA), Governance, Efficiency, Universal Coverage

## Abstract

Cost-effectiveness analysis (CEA) can help countries attain and sustain universal health coverage (UHC), as long as it is context-specific and considered within deliberative processes at the country level. Institutionalising robust deliberative processes requires significant time and resources, however, and countries often begin by demanding evidence (including local CEA evidence as well as evidence about local values), whilst striving to strengthen the governance structures and technical capacities with which to generate, consider and act on such evidence. In low- and middle-income countries (LMICs), such capacities could be developed initially around a small technical unit in the health ministry or health insurer. The role of networks, development partners, and global norm setting organisations is crucial in supporting the necessary capacities.

Baltussen et al^1^ argue against an ever growing volume of one-size-fits-all cost-effectiveness data for supporting healthcare decisions *en route* to universal health coverage (UHC). Instead, they propose what they describe as “evidence-informed deliberative processes” which acknowledge the complex politics involved in making such decisions. Although little empirical evidence exists as to the effectiveness of deliberative processes or their impact on the quality of the decision reached,^2^ it is plausible to suppose that deliberation “as an aid to thought and judgment” and “compared with a ‘closed door’ or ad hoc process” will be “more comprehensive in the relevant issues embraced, more consistent in the way they are embraced and more engaging of the people affected by the outcome.”^3^

Consistent with that belief, national institutions responsible for setting priorities for public spending in healthcare, which use evidence and due process, have emerged in several countries. These are mostly high-income ones – including England and Wales (National Institute for Health and Care Excellence, NICE),^4^ Canada (Canadian Agency for Drugs and Technologies in Health, CADTH)^5^ and the Republic of Korea (National Evidence-based Healthcare Collaborating Agency, NECA),^6^ as well as more recently in low- and middle-income countries (LMICs) including Thailand (Health Interventions and Technology Assessment Program, HITAP^7^) and Brazil (National Committee for Technology Incorporation, CONITEC).^8,9^ These organisations are often known as health technology assessment (HTA) agencies, with “technology” often being much broader than drugs or devices to encompass policies and delivery platforms. Moreover, their function and structure are strongly dependent upon the healthcare system within which they operate. Most explicitly consider cost-effectiveness evidence in their decision-making as well as evidence in the broadest sense, including so-called colloquial evidence on social values and service user perspectives.^3^ As interest in more systematic approaches to allocating resources grows, a global development subfield has evolved including World Health Organization (WHO) CHOICE^10^ and Disease Control Priorities (DCP),^11^ both of which produce global evidence and guidance about “globally” cost-effective health interventions. Baltussen et al^1^ claim that these global trends ignore the local political economy of priority setting and are therefore, unlikely to influence the actual allocation of scarce healthcare resources in LMICs. We agree.

Better decisions about priorities for resource allocation, based on comparative evidence of costs and benefits, and that are feasible and implementable, are becoming increasingly possible in the current move towards UHC.^12^ Many LMICs are experiencing rapid economic growth, with wealthier and more educated populations facing a growing non-communicable disease burden. In combination, these factors are leading to rising demand for quality healthcare, especially in middle-income economies. Although health budgets are increasing year on year, the lack of institutional mechanisms for prioritising services and spending makes attaining UHC all the more challenging. In response, health authorities and payers often set out to define benefits packages,^13,14^ that is, the services and technologies to be covered by public budgets including social health insurance schemes and tax-funded national health services. This often happens through explicit lists, including negative lists of technologies not covered as well as positive lists (also defined as benefits catalogues by Schreyogg et al^13^). Designing and adjusting benefits packages is an example of priority setting, that is, deciding who receives what healthcare at what cost.^8,12^

One way in which things can go wrong is exemplified by the role of the judiciary in priority setting. At least 115 countries worldwide have the right to health enshrined in their constitutions and through legal interpretations.^15,16^ In the absence of corresponding legitimate, transparent and evidence-based priority setting processes, health systems become vulnerable to the country’s judiciary making *ad hoc* decisions on what the system ought to pay for individual patients, often overlooking overall budgetary and other constraints in making such decisions and the resulting impact on the availability of healthcare for the rest of the population. There has been, for example, a mushrooming of court decisions compelling the authorities to provide expensive, often unproven, treatments to specific individuals. A significant share of these cases have been about the delivery of services and technologies already in the benefit packages but which the system has been unable to finance and provide.^15,16^ However, the increasing involvement of the courts in individual treatment decisions and national policies, prioritising human rights of individual patients over affordability for the health system and patients as a whole, can undermine well-intentioned public policy and, at worst, inject further inequalities and inefficiencies into the healthcare system.^17^

To place local values at the heart of decision-making, Baltussen et al^1^ launched an innovative research initiative, REVISE 2020, which openly recognises that “…that priority setting is in reality a value-laden political process in which multiple criteria beyond cost-effectiveness are important, and stakeholders often justifiably disagree about the relative importance of these criteria.”^18^ Our like-minded international Decision Support Initiative (iDSI) (http://www.idsihealth.org/) was established to strengthen in-country institutional and technical capacity together with open participative processes for evidence-informed policy-making.^19^ It takes the form of a collaboration between local policy-makers and other stakeholders for sharing experiences, methods, and knowledge.

## Is the Problem Too Much Cost-Effectiveness Analysis?

Baltussen and colleagues^1^ criticise the promotion of cost-effectiveness analysis (CEA) by global players as the central or even sole criterion in decision-making for all the aforementioned political and value reasons. Yet CEA, as a means of systematically assessing the benefits against the cost of alternative investment options, is in fact not much used by countries’ health authorities in making decisions about real-life public spending in health.^8^ Moving towards a situation where economic evidence is down-valued or even deprioritised over other socially acceptable considerations, with or without deliberation, risks throwing the baby out with the bathwater.

## Should Scientific Value Judgements Be Context-Free?

Global approaches to CEA can hardly be too context-sensitive. Studies done by global players that ignore local contexts but nonetheless presume to advise may undermine local priorities and distort local spending decisions. Without in-country expertise for commissioning, producing and interpreting local data on costs and outcomes,^20^ globally conducted CEA may do more harm than good,^21,22^ particularly if the advice based on such analysis to countries carries weight because of the authority and standing of its authors. Standardised cost-effectiveness decision rules arbitrarily set by global experts with no consideration of local budgetary constraints and opportunity costs,^23^ and based on global generalities such as WHO’s Generalised CEA (explicitly devised to ignore the local realities of current practice through introducing null comparators^24^), serve to promote badly applied cost-effectiveness principles.

CEA is not intrinsically centralising: whether it is depends on the politico-legal structure of the jurisdiction and the corresponding governance arrangements. Further, the “one size fits all” question applies not only at the global versus country level but also within jurisdictions, even the less federalised ones where there are ample opportunities for local priorities to clash with central ones. Countries like India and South Africa exemplify this on a grand scale. In the case of South Africa, there is a relatively small budget for the National Department of Health, and significant devolution of budgets direct to provinces, alongside a sizeable private sector which accounts for more 50% of the spent for servicing less than 20% of the population.^25^ We, therefore, agree with Baltussen et al that at national level there is a critical role for “more generic centrally-led institutionalized processes,” convening the expertise and evidence in the country to inform its priority setting in a way that is relevant. These national processes and decisions not only guide local (subnational) decisions but can be used also to define the scope of local or provincial discretion in following central guidance, providing a transparent means through which differences both of perspective and of material fact can be resolved.

The Lancet Commission on Investing in Health is another example of global advocacy.^26^ It sets out three lists of cost-effective interventions in answer to the question of “what” for UHC.^26^ But, it is not possible (let alone desirable) to determine at the global level what is cost-effective or equitable or otherwise acceptable at country or regional level. The theory may be context-free but its application is not. LMICs are especially vulnerable because they often have very limited capacity to challenge the local applicability of global advice or to conduct independent assessments that take due account of local circumstances. The first question in any respectable guidance for carrying out CEA is, rightly, something like “What is the context?” or “What is the perspective?” for this study.^27^ This is as important a question in LMICs as it is in rich countries. Even at relatively high levels of decision-making, for example, when prioritising health vis-à-vis education, the matter of resource allocation is essentially one for local judgment. After such a high level process, in a well-planned health system of an LMIC committed to UHC, the public budget for health including donor contributions and net of any substitution of donor for national funding^28^ is then available for further prioritisation within health – and will need to respect, amongst other things, the constraints of country-specific donor-set commitments to diseases like HIV and technologies like vaccines, local demographics, local disease burden, local values with respect to equity, local costs of interventions, and informed local judgments about acceptability, feasibility, manageability and speed of implementation in policy and clinical practice.

Such a more nuanced approach is not the norm even in existing international advisory agencies. WHO’s long standing process for updating its Essential Medicines List is another manifestation of limited relevance accorded to context. As a result, aspirational listing of expensive patented pharmaceuticals with little country or subnational guidance on price to ensure value for money, can often be used as a marketing tool by vested interests despite the well-meaning intentions of those access to medicines activists.^29,30^

Criteria for setting priorities are matters of political and social judgment by those who are accountable to the citizens. Technical experts do not have this accountability. We, as iDSI and like Baltussen et al,^1^ subscribe to a deliberative and locally focused approach to decision-making, that is evidence-informed, based on the principles of the iDSI Reference Case for Economic Evaluation.^27^ We support approaches that operate through transparent and independent processes that encourage trust and credibility. Our “one-size-fits-all” solutions are, therefore, set at a very high level and with the possibility of nuancing, for example of the degree of openness and participation, in keeping with such principles as those set out in the accountability for reasonableness framework.^31^ But, what we do not do is to advocate on behalf of particular types of people, categories of disease, or types of intervention or predetermined lists of services. Such matters are to be decided by countries, provinces or districts using the recommended methods and decision-making systems but embodying their own social and political values.^3,31^

## Effective Deliberation Costs Money and Takes Time

Global experts should not dictate the priority that health ought to have over education or other sectors; instead, experts can help local decision-makers develop politically feasible, credible and transparent ways of making such choices. We are making some value judgments here – about decision-making processes rather than the outcome of such processes. Similarly, within health policy, it is not for external experts to decide health priorities. Experts can provide tools and evidence, and, where appropriate, broader policy frameworks. Such is the 2014 WHO resolution on “Health Intervention and Technology Assessment in support of UHC” to support local capacity strengthening and local determination of health priorities.^32^

However, given the limited resources and capacity in many LMICs to create functioning deliberative process in the short run, one can begin with technocratic evidence generation from a small unit in a health ministry or a health insurance agency, aimed at influencing budgets and investment through providing such evidence of trade-offs to decision-makers.^25,33^ Those decision-makers include individuals responsible for making investment decisions at a national or provincial health insurance agency, ministries of health and social security, or ministries of finance. A deliberative process including elements of consultation, transparency, and guarding against vested interests often evolves alongside attempts to generate the needed evidence so that the evidence is more likely to influence decisions,^8^ and both elements (economic evidence and due process) are needed for paving an effective path to influencing resource allocation.

## Concluding Thoughts

CEA matters if countries care about UHC and improving health outcomes.^12,34^ However, CEA that is not based on local evidence is not useful and can even be harmful.^21,22^ A deliberative process taking into account local values and local evidence – including but not limited to CEA evidence – is the holy grail for country empowerment and UHC sustainability and is perhaps the mechanism through which to achieve better spending outcomes in health.^12^ As countries commit to UHC and start to contribute to this commitment financially, there is an opportunity for global partners to align themselves with the principle of context-sensitive evidence as a driver for better decisions, and to support technical, institutional and informational capacity at the local level for achieving this.^25^

It is not the role of global advocacy or technocratic institutions to pre-empt the result of local deliberative processes. The question can be globally put “who or what should get priority?” but the answer must be local. To do otherwise is to disenfranchise national policy-makers and local communities. Their answers may not be those preferred by the global advisers. Why should they be? After all, it is national policy-makers and technocrats, elected by the people or appointed by elected officials, respectively, who will be accountable for their decisions and it is probably these and other local stakeholders who can form the best judgments about what is actually feasible, sustainable and timed rightly for their particular situation. Our responsibility as advisers ought to be to facilitate governments and communities to realize their aspirations, not ours. They decide ends; we can only suggest means.

## Acknowledgments

This work received funding support from the Bill & Melinda Gates Foundation, the Department for International Development (UK), and the Rockefeller Foundation. The funders played no role in the writing of the manuscript. The bulk of this work was completed while the authors KC and RL were employed by NICE International.

## Ethical issues

Not applicable.

## Competing interests

Authors declare that they have no competing interests.

## Authors’ contributions

All authors contributed to drafting of the manuscript. All authors read and approved the final manuscript.

## Authors’ affiliations

^1^Institute of Global Health Innovation, Imperial College London, London, UK. ^2^Department of Economics & Related Studies and Centre for Health Economics, University of York, York, UK. ^3^Center for Global Development, Washington, DC, USA. ^4^School of Public Health, Faculty of Health Sciences, University of the Witwatersrand, Johannesburg, South Africa. ^5^Health Intervention and Technology Assessment Program (HITAP), Nonthaburi, Thailand.
